# The association between the indication for antenatal fetal surveillance and an abnormal result: A retrospective cohort study

**DOI:** 10.1002/pmf2.70282

**Published:** 2026-03-23

**Authors:** Jessica Wu, Hannah S. Foster, Jesse Chittams, Markolline Forkpa, Michael Foster, Nadav Schwartz

**Affiliations:** ^1^ Department of Obstetrics and Gynecology Hospital of the University of Pennsylvania Philadelphia Pennsylvania USA; ^2^ University of Pennsylvania School of Nursing Philadelphia Pennsylvania USA; ^3^ Penn Medicine Women's Health Clinical Research Center University of Pennsylvania Philadelphia Pennsylvania USA; ^4^ Independent statistician Philadelphia Pennsylvania USA

**Keywords:** antenatal testing, high‐risk pregnancy

## Abstract

**Objective:**

Indications for antenatal testing have expanded, but their impact on abnormal test prevalence is unclear. We evaluated factors associated with non‐reactive non‐stress tests (NSTs) and abnormal biophysical profiles (BPPs) in high‐risk pregnancies.

**Study design:**

In this retrospective cohort, we identified all singleton pregnancies that underwent serial antenatal testing with an NST ≥ 28 weeks at four testing sites in an academic health system between January 2022 and May 2023. A BPP was reflexively performed if the NST was non‐reactive. The primary outcome was prevalence of a BPP score <8/10, with the rate of NST non‐reactivity as the secondary outcome. A stepwise Generalized Estimating Equation (GEE) model was used to identify indications associated with these outcomes after adjusting for race, parity, gestational age (GA), and testing site.

**Results:**

Among 19,034 NSTs performed in 2695 patients (mean 7.1 NSTs/patient), only 84 (0.4%) failed to provide a reassuring result (i.e., reactive NST and/or BPP ≥ 8), while 99.6% (18950/19034) had a passing result. The overall NST reactivity rate was 87.3%. Of the 2327 BPPs performed for a non‐reactive NST, 2243 (96.4%) yielded a passing score of ≥8/10. Several testing indications were significantly associated with NST non‐reactivity, including chronic hypertension (aOR, 1.5; CI, 1.2–1.8), fetal growth restriction (FGR) (aOR, 1.4; CI, 1.1–1.6), other fetal/placental complications (aOR, 1.7; CI, 1.2–2.3), obesity, (aOR, 1.3; CI, 1.1–1.5) and hypertensive disorders of pregnancy (HDP) (aOR, 1.3; CI, 1.0–1.6). However, only FGR (aOR, 2.7; CI, 1.5–4.7), vaginal bleeding (aOR, 6.0; CI, 1.4–25), and post‐term (aOR, 8.6; CI, 1.4–51.6) were significantly associated with BPP < 8. For patients in testing for a single indication (79.4% of patients and 73.1% of NSTs), only chronic hypertension (aOR, 1.5; CI, 1.1–2.1) was significantly associated with a higher odds of non‐reactivity, while only post‐term was associated with BPP < 8 (aOR, 7.2; CI, 1.4–35.6).

**Conclusions:**

Our results demonstrate that in the modern‐day clinical environment, only a small fraction (<1%) of fetal tests yield an abnormal result. Several testing indications were more likely to have a non‐reactive NST, but only FGR, vaginal bleeding, and post‐term were more likely to also have an abnormal BPP score < 8. As presenting for serial fetal testing remains a significant barrier for many patients, these data can support data‐informed patient counseling and help clinicians prioritize those at greatest risk. Further research should target investigating downstream perinatal outcomes after abnormal antenatal testing and how changes in testing recommendations impact these outcomes.

## 1 INTRODUCTION

Antenatal testing is routinely recommended for high‐risk patients to reduce the risk of stillbirth. This is based on the concept that fetal hypoxemia and acidosis will be detected as changes in fetal heart‐rate patterns, movement, breathing, and amniotic fluid levels [1, 2]. In fact, a normal antenatal test result—either a non‐stress test (NST), biophysical profile (BPP), or modified BPP (mBPP)—has been associated with a high negative predictive value (>98%–99%) and very low incidence of stillbirth occurring within 1 week of the normal result, providing significant reassurance to patients [3, 4]. However, in the decades since the original fetal surveillance studies were conducted [3, 4, 5], obstetric care and the patient population undergoing testing have evolved considerably.

In clinical practice, there is limited evidence to support standardization of specific testing indications and protocols. Furthermore, prospective research exploring the impact of different approaches to reduce stillbirth would require randomization of tens of thousands of patients to different surveillance regimens to allow for robust conclusions. Acknowledging these challenges, the American College of Obstetricians and Gynecologists (ACOG) published a Committee Opinion regarding antenatal fetal surveillance, which included an extensive list of risk factors associated with an increased risk of stillbirth that should be considered for antenatal testing after a shared decision‐making discussion with the patient [2]. Standard indications that are universally recommended for antenatal testing based on a more than twofold increased relative risk of stillbirth include fetal growth restriction (FGR), multiple gestations, maternal hypertension on medications, maternal diabetes, obesity, and poor obstetric history. Other indications, such as complicated multiple gestations, fetal anomalies, poorly controlled maternal medical conditions, or advanced maternal age, can be recommended for individualized timing and frequency of antenatal testing for each patient [2]. To help inform this counseling, we sought to explore the relationship between different testing indications and the odds of an abnormal fetal test result.

## 2 MATERIALS AND METHODS

We performed a retrospective cohort study of all singleton pregnancies that underwent serial (once or twice weekly) antenatal testing at ≥28 weeks’ gestation and subsequently delivered at one of three hospitals within our institution's health system between January 2022 and May 2023. The primary testing strategy in our institution is to begin with an NST or modified BPP (i.e., NST plus amniotic fluid measurement), with a full BPP being performed in cases where the NST was non‐reactive. Tests performed for multifetal gestations, at gestational ages <28 weeks, or whose testing indications or delivery outcomes were not available were excluded. We also excluded BPPs documented incidentally that were not performed for a non‐reactive NST.

Data on demographics, indication(s) for antenatal testing, gestational age at testing visit, and testing result were collected from the electronic medical record. For patients who had multiple indications for antenatal testing, these were considered as multiple equivalent exposures. NSTs were defined as reactive or non‐reactive, and BPPs were defined as passing (score ≥ 8) or abnormal (score < 8/10). The primary outcome was abnormal BPP (<8/10), and the secondary outcome was NST non‐reactivity.

Descriptive statistics were calculated for patient demographics, testing indications, and gestational age at the time of test. A Generalized Estimating Equation (GEE) using a stepwise variable selection mechanism was used, as each patient could have multiple correlated data points (i.e., multiple NST visits per patient). The stepwise GEE models were used to explore adjusted associations between patient characteristics, testing indication, and our study outcomes. The alpha level for entry was *p* < 0.15, and the alpha level to remain in the model was *p* < 0.15. An alpha level of *p* < 0.05 determined statistical significance. Analyses were performed using R computing software.

This study was conducted using retrospective data and did not involve direct interaction with human participants. In accordance with Institutional Review Board (IRB) guidelines and national regulations, the requirement for informed consent was waived as the research involved minimal risk to subjects and included only previously collected, de‐identified data. All study procedures were approved by the University of Pennsylvania Institutional Review Board (IRB#853416).

## 3 RESULTS

**FIGURE 1:**
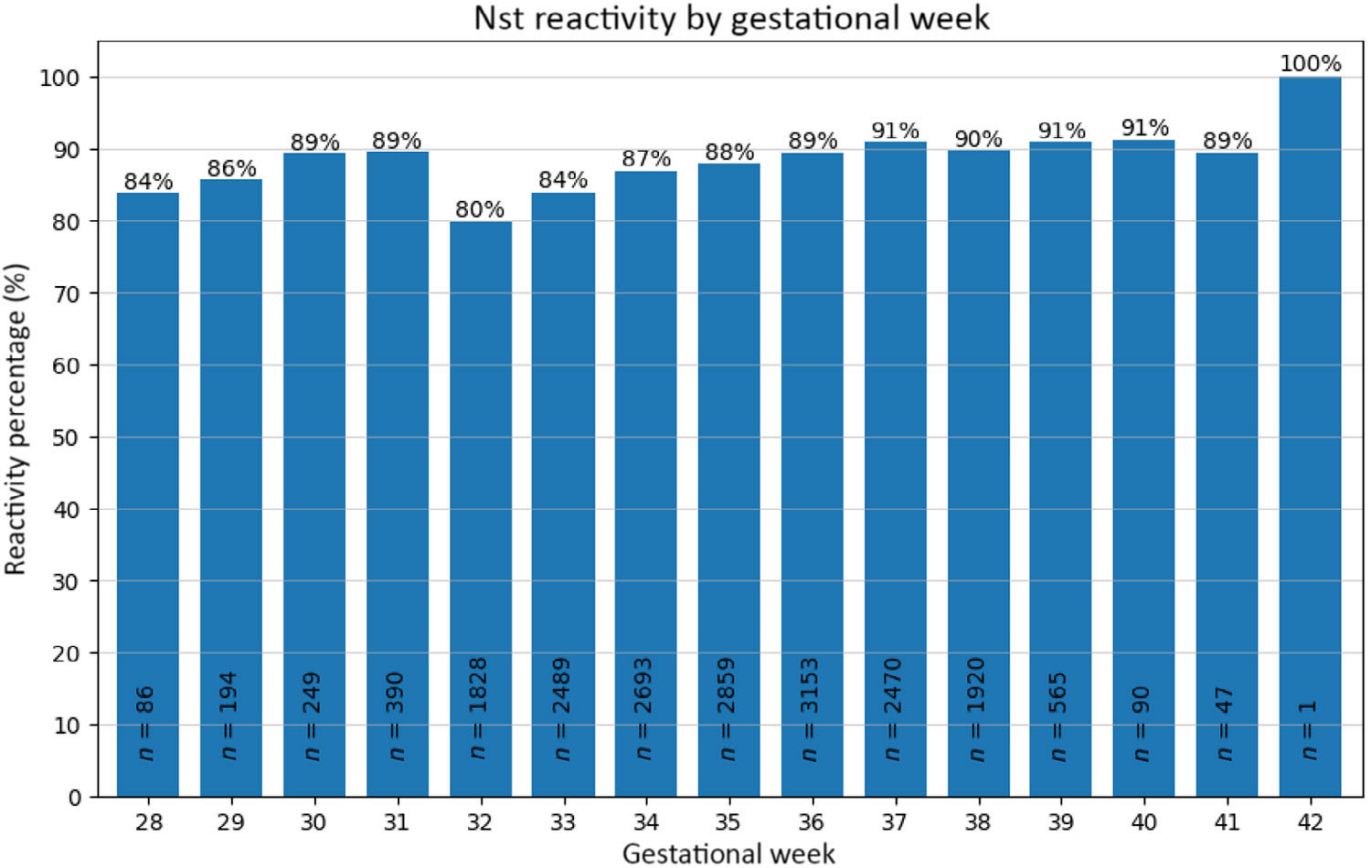
Non‐stress test (NST) reactivity by gestational age.

**FIGURE 2:**
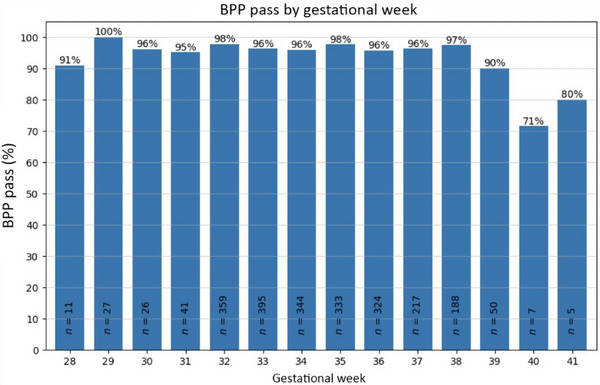
BPP score > 8 by gestational age. BPP, biophysical profile.

A total of 20,887 testing visits and 2717 patients were included. Of these, 19,034 were NSTs performed in 2695 patients, with a mean of 7.1 NSTs per patient. The remaining 1853 tests were isolated BPPs, either incidental or without associated NST, and were excluded. Mean maternal age was 32.6 years, with mean body mass index (BMI) of 31.2 kg/m^2^. The overall NST reactivity rate was 87.3%, with Figure 1 showing reactivity rates by gestational age. The odds of non‐reactivity were higher for patients with chronic hypertension, fetal/placental complications, obesity, fetal growth restriction (FGR), and hypertensive disorders of pregnancy (HDP) (Table 1). The Black race had increased odds of non‐reactivity (17.8%, aOR 2.18), and non‐reactivity varied significantly by testing site (from 6.0% to 15.5%), even after controlling for indication. 96.4% (2243/2327) of BPPs after a non‐reactive NST had a score of 8/10. Figure 2 shows the prevalence of passing BPP scores by gestational age. FGR, vaginal bleeding, and post‐term were the only indications significantly associated with BPP < 8 (Table 1). Overall, 99.6% of NSTs in our cohort were either reactive or had a normal BPP.

**TABLE 1:** Prevalence of NST non‐reactivity and BPP < 8 by testing indication. Adjusted odds ratios are presented for indications significantly associated with increased odds of NST non‐reactivity and BPP < 8.

Testing indication^a^	NST non‐reactivity [*n*/*N* (%)]	aOR (95% CI)	BPP < 8 [*n*/*N* (%)]	aOR (95% CI)
Fetal growth restriction (FGR)	717/4749 (15.1)	1.4 (1.1–1.6)	34/696 (4.9)	2.7 (1.5–4.7)
Obesity	649/4282 (15.2)	1.3 (1.1–1.5)	21/614 (3.4)	NS
Advanced maternal age (AMA)	391/4100 (9.5)	NS	18/368 (4.9)	NS
Gestational diabetes‐A2 (GDMA2)	291/2550 (11.4)	NS	12/279 (4.3)	NS
Chronic hypertension (cHTN)	443/2516 (17.6)	1.5 (1.2–1.8)	11/430 (2.6)	NS
Hypertensive disorders of pregnancy (HDP)	184/1274 (14.4)	1.3 (1.0–1.6)	4/177 (2.3)	NS
Pregestational diabetes	204/1263 (16.2)	NS	8/195 (4.1)	NS
Thromboembolic disease	124/1036 (12.0)	NS	3/122 (2.5)	NS
Other maternal medical conditions^b^	98/848 (11.6)	NS	0/96 (0.0)	5.2 × 10^−18^ (3.0 × 10^−18^–8.9 × 10^−18^)
Other fetal/placental indications^c^	150/798 (18.8)	1.7 (1.2–2.3)	9/147 (6.1)	NS
Poor obstetric history	108/718 (15.0)	NS	2/104 (1.9)	NS
Vaginal bleeding (with previa/abruption)	31/373 (8.3)	NS	4/30 (13.3)	6.0 (1.4–25.0)
Cholestasis	31/345 (9.0)	NS	2/31 (6.5)	NS
Decreased fetal movement	38/328 (11.5)	NS	1/38 (2.6)	NS
Post‐term pregnancy	11/113 (9.7)	NS	2/10 (20.0)	8.6 (1.4–51.6)

Abbreviations: aOR, adjusted odds ratio; BPP, biophysical profile; CI, confidence interval; NS, not significant; NST, non‐stress test.

^a^ NSTs may have been performed for > 1 indication(s).

^b^ Maternal medical conditions included cardiopulmonary disease, autoimmune disease, and hematologic disorders.

^c^ Fetal/placental indications included oligohydramnios, polyhydramnios, and fetal anomalies.

An additional sub‐analysis was also performed on patients who underwent testing for only a single indication. Overall, 79.4% (*n* = 2140) of patients and 73.1% (*n* = 13923) of NSTs had only a single indication for testing. This sub‐group had a similar rate of NST reactivity (88.4%). Overall, only chronic hypertension (aOR, 1.5; CI, 1.1–2.1) was associated with a higher odds of non‐reactivity, whereas patients in testing for advanced maternal age had a lower odds (aOR, 0.7; CI, 0.6–0.9). The Black race also had increased odds of non‐reactivity in this sub‐group (aOR 2.2), and non‐reactivity continued to vary significantly by testing site. For BPPs performed after a non‐reactive NST, 96.5% (1510/1564) had a score of ≥8/10. Thus, only 0.4% (54/13923) of testing visits performed for a single indication resulted in an equivocal or abnormal BPP. Post‐term was the only indication significantly associated with BPP < 8 (aOR, 7.2; CI, 1.4–35.6) in this sub‐group.

## 4 DISCUSSION

### 4.1 Principal findings

Our study investigated the clinical conditions associated with abnormal antenatal testing and demonstrated the exceedingly low prevalence of abnormal fetal testing in a high‐risk patient population undergoing serial fetal surveillance. In fact, the rate of a non‐reactive non‐stress test with an abnormal BPP was only 0.4%. While several indications were associated with a higher likelihood of a non‐reactive NST, only FGR, vaginal bleeding, and post‐term were associated with an increased rate of abnormal BPP score < 8. When further narrowing testing visits to those performed for only a single indication, less than 0.5% of testing visits resulted in an abnormal BPP. Only chronic hypertension was associated with a non‐reactive NST, with only post‐term being associated with an increased rate of abnormal BPP < 8.

### 4.2 Results and implications

Antenatal testing was shown to be associated with a reduced risk of perinatal mortality and stillbirth in various high risk populations. As clinical practice and patient populations evolved in the decades since the initial studies were performed, significant variability in the fetal surveillance indications emerged. Our results provide new data that inform this counseling by demonstrating the low overall prevalence of abnormal testing results as well as the few indications that are significantly associated with an increased risk of having a non‐reactive NST of abnormal BPP < 8. This can potentially reassure anxious patients concerned about the well‐being of their fetus after being recommended for antenatal surveillance to mitigate their risk for stillbirth. Furthermore, when specifically considering only patients in testing for a single indication, these associated indications are even fewer, which could provide additional reassurance to patients in this situation.

However, while the goal of reducing stillbirth risk is paramount, the need to present for serial appointments can pose a significant burden for patients and institutions alike, especially in lower resource settings. There are no large clinical trials to dictate the optimal frequency or protocol for antenatal testing [1]. Given the large range of testing indications, it can reasonably be assumed that the clinical stillbirth risk lies on a spectrum based on indication. In addition, antenatal testing is also not without risks, such as that of false positives and subsequent iatrogenic prematurity or cesarean delivery [4]. Thus, our data may be used by clinicians to inform the shared decision‐making with patients regarding fetal surveillance. For patients who have significant barriers to presenting for frequent in‐person testing appointments, or in under‐resourced settings, where the availability of technology and providers to allow for antenatal testing may be limited, these results may also help stratify patients to prioritize those at the highest risk of identifying an abnormal testing result.

Our results cannot be used to make specific recommendations for modifying testing protocols, as we are not comparing pregnancy outcomes for different testing strategies. However, we would note that there are several indications for which ACOG provides a range of frequency options (e.g., once or twice weekly) or suggests that testing frequency should be “individualized.” [2] It could be that our data can help inform the shared decision‐making between patients and providers to individualize the testing regimen for patients based on their indications and the associated risk of abnormal test results.

### 4.3 Strengths and limitations

Our study included over 20,000 testing visits, providing a large sample size to explore the associations between various testing indications and abnormal testing results. We also included four different sites within our academic institution. However, this study is still limited by its retrospective nature and the potential presence of unidentified confounders. In addition, we focused on exploring the relationship between testing indication and abnormal fetal test results, and data on downstream delivery/perinatal outcomes were not collected or analyzed for this study. Therefore, no conclusions can be made regarding pregnancy outcomes and whether abnormal fetal test results indicated true or false positives, which was beyond the scope of this study. Moreover, the inability to have a proper control group with similar risk factors but that did not undergo testing precludes any assessment of the potential risk reduction achieved by fetal testing for specific indications. Further research should focus on investigating how abnormal fetal tests in different testing indications impact downstream delivery and perinatal outcomes.

## 5 CONCLUSION

The prevalence of an abnormal fetal testing result among high‐risk, singleton pregnancies undergoing serial antenatal testing is exceedingly low (<1%). While several indications are associated with an increased risk of a non‐reactive NST, <5% of subsequent BPPs return an abnormal result (<8). In fact, of the numerous indications for antenatal testing, only FGR, vaginal bleeding, and post‐term pregnancy were at increased odds of an abnormal BPP (>8). As presenting for serial fetal testing remains a significant barrier for many patients, the results of this study can be used by clinicians during patient counseling to support data‐informed fetal surveillance regimens driven by individualized patient risk and shared decision‐making.

## CONFLICT OF INTEREST STATEMENT

Nadav Schwartz has served as an advisor and consultant for Nuvo Group, a company that manufactures a remote pregnancy monitor. The other authors have no conflicts of interest to report.

## FUNDING INFORMATION

The authors received no specific funding for this work.

## ETHICS STATEMENT

This study was approved by the University of Pennsylvania Institutional Review Board (IRB#853416).

## Data Availability

The datasets generated and analyzed for this study are available from the corresponding author on reasonable request.
